# A systematic review of present and future pharmaco‐structural therapies for hypertrophic cardiomyopathy

**DOI:** 10.1002/clc.24207

**Published:** 2024-01-03

**Authors:** Mariem A. Sawan, Sindhu Prabakaran, Melroy D'Souza, Omid Behbahani‐Nejad, Matthew E. Gold, Byron Robinson Williams, Ozlem Bilen

**Affiliations:** ^1^ Division of Cardiology Emory University School of Medicine Atlanta Georgia USA; ^2^ Department of Internal Medicine Emory University School of Medicine Atlanta Georgia USA

**Keywords:** cardiomyopathy, hypertrophic cardiomyopathy, left ventricular outflow tract obstruction, non‐obstructive hypertrophic cardiomyopathy, obstructive hypertrophic cardiomyopathy, sudden death

## Abstract

Hypertrophic cardiomyopathy (HCM) is a common contemporary, treatable, genetic disorder that can be compatible with normal longevity. While current medical therapies are ubiquitous, they are limited by a lack of solid evidence, are often inadequate, poorly tolerated, and do not alter the natural disease course. As such, there has long been a need for effective, evidence‐based, and targeted disease‐modifying therapies for HCM. In this review, we redefine HCM as a treatable condition, evaluate current strategies for therapeutic intervention, and discuss novel myosin inhibitors. The majority of patients with HCM have elevated left ventricular outflow tract gradients, which predicts worse symptoms and adverse outcomes. Conventional pharmacological therapies for symptomatic HCM can help improve symptoms but are often inadequate and poorly tolerated. Septal reduction therapies (surgical myectomy and alcohol septal ablation) can safely and effectively reduce refractory symptoms and improve outcomes in patients with obstructive HCM. However, they require expertise that is not universally available and are not without risks. Currently, available therapies do not alter the disease course or the progressive cardiac remodeling that ensues, nor subsequent heart failure and arrhythmias. This has been regarded as an unmet need in the care of HCM patients. Novel targeted pharmacotherapies, namely cardiac myosin inhibitors, have emerged to reverse key pathophysiological changes and alter disease course. Their favorable outcomes led to the early Food and Drug Administration approval of mavacamten, a first‐in‐class myosin modulator, changing the paradigm for the pharmacological treatment of HCM.

## Graphical abstract

Hypertrophic cardiomyopathy (HCM) is an increasingly recognized genetic condition that is accompanied by a heterogeneous phenotype. The presence of outflow tract obstruction carries an increased risk for the development of heart failure, arrhythmias, and sudden cardiac death. Here, we review conventional and novel disease‐modifying therapies for HCM.

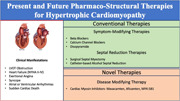

**Abbreviations**
ACC/AHA/ESC: American College of Cardiology/American Heart Association/European Society of Cardiology
AF: atrial fibrillation
ASA: alcohol septal ablation
BB: beta‐blocker
CCB: calcium channel blocker
FDA: Food and Drug Administration
HCM: hypertrophic cardiomyopathy
HF: heart failure
KCCQ: Kansas City Cardiomyopathy Questionnaire
LV: left ventricle
LVEF: left ventricular ejection fraction
LVOT: left ventricular outflow tract
LVOTG: left ventricular outflow tract gradient
LVOTO: left ventricular outflow tract obstruction
nHCM: non‐obstructive hypertrophic cardiomyopathy
NYHA: New York Heart Association
oHCM: obstructive hypertrophic cardiomyopathy
pVO_2_
: peak oxygen consumption
SCD: sudden cardiac death
SM: surgical myectomy
SRT: septal reduction therapies
VSD: ventricular septal defect

## 1 INTRODUCTION

### 1.1 HCM is a common contemporary treatable genetic disorder that can be compatible with normal longevity

Hypertrophic cardiomyopathy (HCM) is the most common inherited heart disease, with an estimated clinical prevalence that approaches 1:200 in the general population and is responsible for significant morbidity and mortality in patients of all ages.^1^ It is a heterogeneous disease with diverse disease‐causing mutations, clinical presentation, and outcomes.^1^, ^2^, ^3^ Patients with HCM are commonly asymptomatic or are minimally symptomatic, which explains why the disease is often underdiagnosed.^4^ However, for many, HCM has the potential to cause progressive dyspnea, angina, heart failure (HF) with or without left ventricular (LV) systolic dysfunction, atrial fibrillation (AF), and sudden cardiac death (SCD), the latter being the most feared complication.^3^, ^5^ HCM was first defined over 60 years ago. With the advantage of contemporary treatments that are personalized to target disease complications, including implantable cardioverter‐defibrillators, annual disease‐related mortality has substantially dropped.^6^

Today, in addition to SCD prevention, clinical management of HCM focuses mainly on symptom relief. Medications with negative inotropic properties (i.e., beta‐blockers [BB] and non‐dihydropyridine calcium channel blockers [CCB]) are first‐line therapy in all HCM subtypes, while disopyramide is used as a second‐line agent in those with left ventricular outflow tract obstruction (LVOTO). For patients with obstructive HCM (oHCM) and drug‐refractory symptoms, lowering outflow tract gradients through interventions for septal reduction can provide symptomatic relief.^7^

## 2 HYPOTHESIS

While current medical therapies are ubiquitous, they are limited by a lack of solid evidence, are often inadequate, poorly tolerated, and do not alter the natural disease course. As such, there has long been a need for effective, evidence‐based, and targeted disease‐modifying therapies for HCM. In this review, we redefine HCM as a treatable condition, evaluate current strategies for therapeutic intervention, and discuss novel myosin inhibitors.

## 3 METHODS

All of the authors took the independent responsibility of performing a thorough literature review and selecting landmark clinical trials related to HCM. The authors cross‐referenced their findings and created a comprehensive list of landmark clinical trials for further analysis as part of this systematic review. Clinical trials of therapies that failed to proceed to the next phase of the study were excluded from this comprehensive list. Thus, the studies referenced in this manuscript include the most up‐to‐date active clinical trials with the hopes of advancing the field of HCM.

## 4 RESULTS

### 4.1 Anatomic phenotypes and clinical presentation: Obstruction predicts worse symptoms, adverse outcomes and becomes a therapeutic target

HCM can be defined as LV hypertrophy (LVH) at any site with maximum LV thickness ≥15 mm in the absence of abnormal loading conditions. Milder hypertrophy may be diagnostic if associated with a family history of HCM, typical outflow obstruction, or abnormal ECG patterns. Various patterns of LVH have been described, that is, diffuse hypertrophy, and asymmetric involvement of ventricular septum, anterior wall, or apical chamber.

Two‐thirds of patients diagnosed with HCM have LVOTO at rest or with provocation.^8^ LVOTO is defined as dynamic LV outflow pressure ≥30 mmHg, while pressures ≥50 mmHg are considered hemodynamically significant.^9^, ^10^ LVOTO strongly predicts future HF progression.^11^ In patients with a hemodynamically significant obstruction (i.e., left ventricular outflow tract gradient [LVOTG] ≥50 mmHg) and pharmacologic refractory HF‐related symptoms, subsequent therapeutic options are septal reduction therapies (SRTs) (i.e., surgical myectomy [SM] and alcohol septal ablation [ASA]). However, cardiac transplant may be the only option for HCM patients without LVOTO (non‐obstructive HCM [nHCM]) and those with end‐stage disease, characterized by advanced HF and restrictive ventricular filling.^12^ Hence, delineating ventricular anatomy and hemodynamics is essential to guide management.

### 4.2 Conventional pharmacological therapies for symptomatic HCM can help improve symptoms, but are often inadequate, poorly tolerated, and do not alter the natural disease course

Beta‐blockers have been a cornerstone of HCM therapy since their introduction for this purpose in 1964.^13^ They are currently recommended as first‐line agents in symptomatic patients both with and without resting obstruction.^7^ The beneficial effects of BB in HCM are thought to be mediated by several different mechanisms, including sympathetic modulation of heart rate, improved ventricular relaxation with increased time for diastolic filling, and reduced electrical excitability.^14^

Once it was recognized that the severity of LVOTO may be influenced by the degree of myocardial contractility, Harrison et al. were the first to study BB in 10 patients with HCM in 1964. They found that nethalide (an adrenergic BB) had little effect on the degree of obstruction at rest, but prevented the increase in LVOTO that was observed during exercise.^15^ Subsequently, Cohen and Braunwald discovered that propranolol decreased myocardial oxygen requirements and improved anginal symptoms.^16^ The only prospective randomized, placebo‐controlled BB trial was conducted by Dybro et al.^17^ In 29 patients with oHCM, metoprolol reduced the LVOTG at rest by 33 ± 25 mmHg. Similarly, metoprolol reduced the LVOTG with Valsalva provocation by 66 ± 48 mmHg and reduced the LVOTG with exercise by 47 ± 36 mmHg. This occurred in addition to concomitant symptom relief and improvements in quality of life scores.^17^ It is currently unknown if any specific BB is superior to others with regard to their selectivity, pharmacodynamics, or antiarrhythmic properties. In general, BB has shown to be most effective in relieving symptoms of dynamic, exercise‐induced LVOTO, and is less effective when severe obstruction is present at rest.^14^, ^17^, ^18^ The 2020 American Heart Association (AHA)/American College of Cardiology (ACC) HCM guidelines suggest that BB should be titrated to a dose where there is symptom benefit but not declare a failure of beta‐blockade until there is demonstrated physiologic evidence of beta‐blockade, such as bradycardia.^7^ Despite its everyday clinical use as a first‐line therapy in patients with oHCM and nHCM, the common side effects of BB (i.e., bradycardia, erectile dysfunction, fatigue, weight gain) may limit its use, particularly in the young and elderly patient population.

*Non‐dihydropyridine calcium channel blockers* have also alleviated symptoms in a subset of patients. The 2020 AHA/ACC HCM guidelines recommend CCB and BB for first‐line use in patients with obstructive and non‐obstructive HCM.^7^ The beneficial effects of CCBs are likely mediated through their negative inotropic and chronotropic effects, leading to prolonged LV filling time and improved flow towards the LV's subendocardial layers, resulting in reduced myocardial oxygen demand.^19^ Verapamil should be used cautiously in patients with severe obstruction, given vasodilatory properties that may contribute to the worsening of the intracavitary gradient.^20^, ^21^ Diltiazem is less studied but has been shown to improve measures of diastolic performance and myocardial ischemia.^20^, ^21^ In patients unable to tolerate BB; CCB can be considered but should be used cautiously in those with severe LVOTO, elevated pulmonary capillary wedge pressure, and low systemic blood pressure, as their hypotensive effects can trigger worsening obstruction and precipitate pulmonary edema.^5^

*Disopyramide* can provide symptomatic relief in patients with oHCM. The 2020 AHA/ACC HCM guidelines recommend using disopyramide as an adjunct in patients who remain symptomatic despite the use of BB and CCB.^7^ Disopyramide is a Class IA antiarrhythmic; a sodium channel blocker with potent negative inotropic effects mediated by action potential prolongation. Decreasing LV contractility through early LV ejection flow deceleration allows for the relief of obstruction. Disopyramide has a more substantial gradient‐lowering effect than other negative inotropes for oHCM. Its safety and efficacy in HCM patients have been previously demonstrated.^22^ Most recently, the safety of disopyramide initiation was studied in the outpatient setting.^23^ Of the 168 outpatient participants started on disopyramide, no significant cardiac events occurred within 3 months. However, 23% developed side effects and 11% stopped the drug because of these side effects. These side effects include QTc prolongation and anticholinergic effects, which often prohibit its long‐term use. Anticholinergic effects can be managed with pyridostigmine; however, it is often poorly tolerated.^7^ In addition to the unfavorable side effect profile, disopyramide's use is limited among clinicians due to a lack of familiarity and hesitation to use a Class IA antiarrhythmic in patients with LVH. In addition, disopyramide's antiarrhythmic properties are advantageous in HCM patients with AF.^24^ It is generally suggested that disopyramide should be coupled with a BB or CCB as disopyramide monotherapy is potentially harmful due to the enhancement of atrioventricular conduction and, consequently, increased ventricular rates during AF.^10^ Ultimately, disopyramide can be added in combination with either BB or CCB for patients with oHCM who have persistent symptoms.^7^

### 4.3 Septal reduction therapies can safely and effectively help patients with refractory symptoms and improve outcomes, but require expertise that is not universally available

In patients with oHCM who remain severely symptomatic despite optimal medical therapy, SRT, when performed by experienced operators in comprehensive centers, is effective for relieving LVOTO. Both SM and ASA can provide improvement in the New York Heart Association (NYHA) class, short‐term, and long‐term mortality. Comparing ASA to SM, several meta‐analyses demonstrated ASA having higher rates of permanent pacemaker (PPM) implantation and reintervention. In contrast, SM is associated with greater rates of peri‐procedural mortality and stroke.^25^, ^26^, ^27^, ^28^, ^29^, ^30^, ^31^, ^32^, ^33^, ^34^

The first septal myectomy was performed in 1958.^35^ Surgery involves resection of the septum through an aortic incision, which results in the widening of left ventricular outflow tract (LVOT), reduction in systolic anterior motion, mitral regurgitation and LVOTO. Multimodality imaging studies showed a reversal of LV and LA remodeling following surgery.^36^, ^37^, ^38^ Data from experienced centers with high surgical volumes have demonstrated low surgical mortality rates at <1%. Over 90% of patients demonstrated ≥1 NYHA functional class improvement, and >75% had complete symptom resolution. Surgical excision aims to obtain laminar flow in LVOT, reduces postoperative gradients to nil, and achieves permanent relief of LVOTO. Long‐term survival after myectomy is similar to the general HCM population.^39^ Complications of septal myectomy include ventricular septal defects (VSDs) (1%) and complete heart block requiring PPM implantation (3%–10%).^26^, ^28^, ^40^, ^41^, ^42^, ^43^, ^44^, ^45^

Percutaneous ASA was first reported in 1995 and has been endorsed by the 2020 ACC/AHA guidelines as an alternative to surgery for reducing gradients and drug‐refractory symptoms in patients who are deemed to be a high surgical risk.^7^, ^39^ ASA is a minimally invasive, catheter‐based procedure involving the injection of dehydrated ethanol into septal perforators to create a targeted myocardial infarction in the area of the hypertrophied septum, reducing the intracavitary gradient and relieving obstruction.^46^ Before ASA, coronary angiography is performed to delineate coronary anatomy, and a temporary transvenous pacemaker is placed for pacing during the procedure until native conduction is re‐established. There can be anatomical variation in the location of the septal perforator. In cases in which the area supplied by a septal perforator is less clear, contrast echocardiography can be utilized to visualize the perfusion area before alcohol injection. Continuous gradient monitoring during the procedure can demonstrate an immediate reduction in LVOTG, thereby indicating a favorable response. While the initial decline in LVOTG is likely representative of myocardial stunning, over time, there is a gradual reduction in the gradient correlated to delayed thinning of the basal septum.^47^ Unlike SM, which can be performed at any wall thickness, ASA is preferred for those only with >18 mm and <30 mm septum. Also, in contrast to SM, LVOTG reduction is less consistent, and repeat ablations might be required in up to 20% of patients.^30^ Resulting transmural scars can be arrhythmogenic and increase mortality.^48^ Furthermore, the inability to address concomitant anatomic abnormalities of the mitral valve or LVOT and higher PPM requirements make this a less favorable choice. Nonetheless, several advantages are present, including faster recovery time and avoidance of an open sternal incision.^5^, ^7^, ^10^, ^25^, ^30^, ^39^, ^49^

It is important to note that outcomes for these invasive procedures are related to procedural volumes. A nationwide inpatient database included 6386 patients who underwent SM and 4862 who underwent ASA. 60% of institutions performed <10 SM over 9 years, and in‐hospital mortality was as high as 15.6% in the lowest surgical volume tertile. Similarly, 70% of institutions performed <10 ASA during the study period. Even in the center at the highest surgical volume tertile, in‐hospital death, need for permanent pacing, stroke, bleeding, and acute renal failure were not negligible (3.8%, 8.9%, 1.9%, 1.7%, and 9.4%, respectively).^50^ Many patients worldwide with LVOTO are managed in institutions with limited procedural expertise, and many patients have limited access to large‐volume centers with experienced operators. For such patients, pharmacotherapy continues to be the mainstay for HCM management.

### 4.4 Unmet needs: Progressive remodeling, prevalent heart failure, and arrhythmia (Figure 1)

Our understanding of HCM has deepened over the past 50 years. Although mortality has decreased, HF and arrhythmia continue to develop due to disease progression. While current therapies can improve symptoms in a subset of patients, they do not address the underlying disease pathophysiology. Data from the Sarcomeric Human Cardiomyopathy Registry (SHARE) on 4591 patients showed a substantial cumulative burden of HCM‐related complications, including those with LVOTO undergoing SRT, dominated by HF and AF occurring many years after diagnosis.^51^ Even with complete surgical relief of obstruction with myectomy, up to 40% of patients fail to improve their exercise peak oxygen consumption (pVO_2_).^52^ This suggests factors other than obstruction, such as impaired diastolic filling, determine exercise capacity in HCM.^53^ This unmet need led to molecular, cellular, and human genetics studies, which resulted in the discovery of disease‐modifying therapies, namely cardiac myosin inhibitors (CMI).

**Figure 1:**
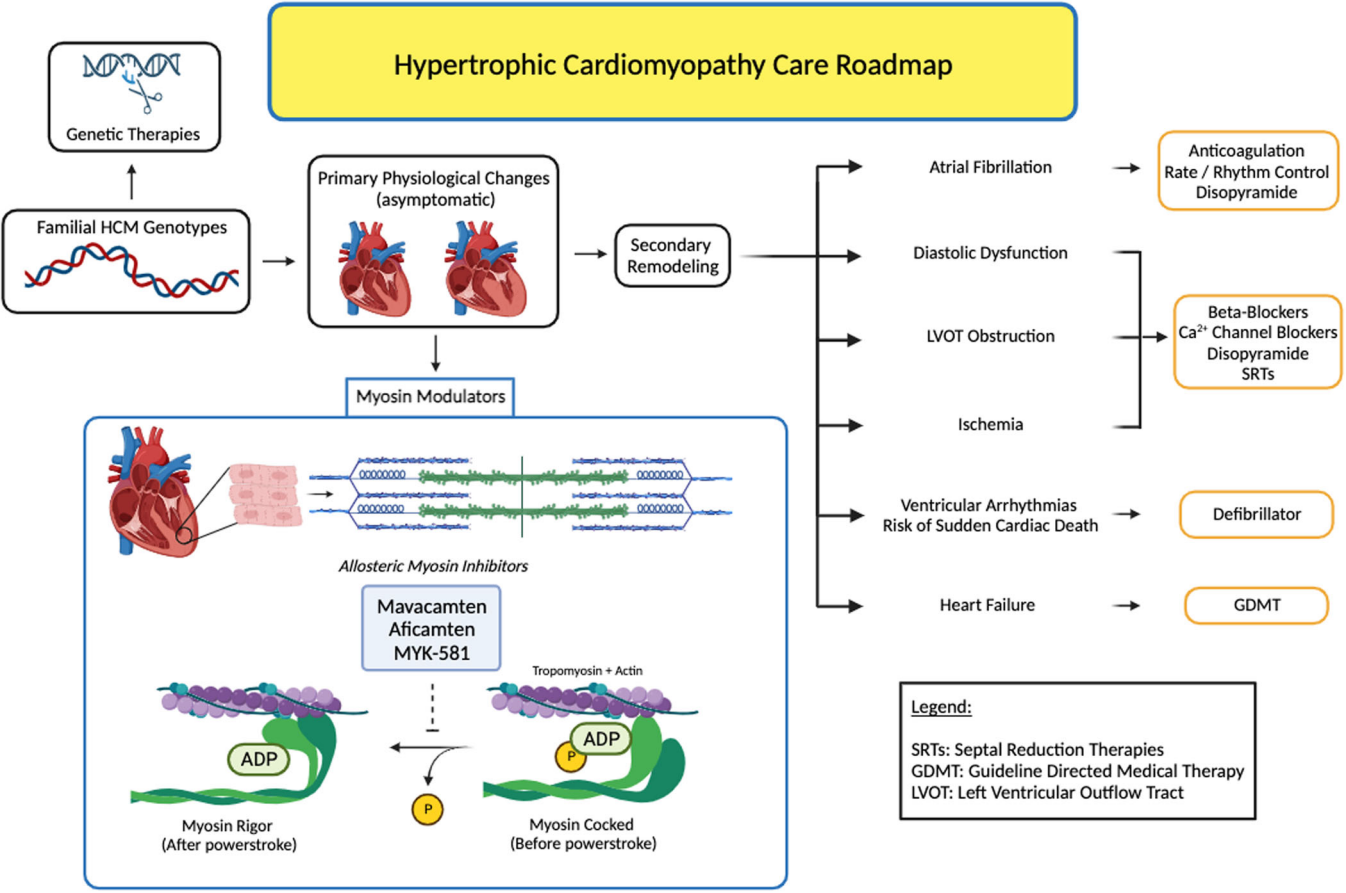
Hypertrophic cardiomyopathy care roadmap. This figure illustrates the foundation of hypertrophic cardiomyopathy originating from genetics, the consequences of secondary remodeling, and therapeutic options before and after secondary remodeling occurs.

Mutations of the cardiac sarcomere are the underlying cause of HCM. These mutations lead to the excessive cross‐bridging of myosin heads with actin and increased cardiac contractility.^54^ This is thought to represent the primary pathophysiological abnormality ultimately generating the HCM phenotypes, from myocardial hypertrophy to impaired relaxation, LVOTO, ischemia, scar formation, and arrhythmias. Inhibiting the myosin ATPase via CMIs has been shown to reduce the number of available myosin heads, resulting in a return to the normal contractile state, relief of LVOTO, decreased myocardial wall stress, and improved relaxation.^55^ There are two main CMIs currently in various stages of development, MyoKardia has developed mavacamten (formerly MY461), MYK‐224 and MYK‐581, while cytokinetics has developed aficamten (formerly CK‐274) (Table 1).

**Table 1:** Clinical trials focusing on obstructive and non‐obstructive hypertrophic cardiomyopathy, their study design, and brief summaries are noted.

Trial	Design	Study population	Intervention	*N*	Duration	Aim	Main findings	Adverse findings
Subtype: Obstructive hypertrophic cardiomyopathy								
PIONEER‐HCM: Cohort A	Prospective, Phase II, open‐label, clinical trial	NYHA Class II and III. Resting LVOTG of ≥30 or ≥50 mmHg.	*Mavacamten* 10 or 15 mg daily	11	12 weeks	Effect of Mavacamtem on myocardial contractility and LVEF.	Reduction in resting and postexercise LVOTG, improvement in pVO_2_, and reduction in serum NT‐proBNP.	Reduction in resting LVEF: 15%. The LVEF returned to baseline levels 4 weeks after treatment.
PIONEER‐HCM: Cohort B	Prospective, Phase II, open‐label, clinical trial	NYHA Class II & III. Resting LVOTG ≥30 or ≥50 mmHg postexercise.	*Mavacamten* 5 mg daily with beta‐blockers allowed	10	12 weeks	Dose‐dependent response of Mavacamten on LVOTG.	Reduction in resting and postexercise LVOTG, improvement in pVO_2_ and serum NT‐proBNP.	Reduction in resting LVEF: 6%. The LVEF returned to baseline levels 4 weeks after treatment.
PIONEER‐OLE	Prospective, open‐label, clinical trial	Patients who completed parent PIONEER‐HCM trials.	*Mavacamten* starting dose 5 mg/day; titration at Week 6 to an individualized dose (5, 10, or 15 mg)	12	3 years	Change in LVEF, LVOTG, NYHA Class, NT‐proBNP, drug concentration, and safety.	Persistent improvements in LVOTG, *E*/*e*′, LAVi, NT‐proBNP, IVS, and NYHA classification at 1 year.	No drug related SAEs.
EXPLORER‐HCM	Prospective, Phase III, double‐blinded, randomized, multicenter clinical trial	NYHA II or III. LVOTG ≥50 mmHg.	*Mavacamten* 2.5–15 mg daily dosed to achieve LVOTG <30 mmHg and serum concentration of 350–700 ng/mL	251	30 weeks	The primary endpoint: ≥1.5 mL/kg/min increase in pVO_2_, ≥1 NYHA class reduction, or a ≥3 mL/kg/min pVO_2_ increase without worsening NYHA status.	37% of 123 patients on Mavacamten vs. 17% of 128 on placebo met the primary endpoint. Greater improvement of LVOTG, symptom scores (KCCQ‐CSS, HCMSQ‐SoB) and greater reduction in serum NT‐proBNP and hs‐cTnI concentrations were noted in the Mavacamten group.	Reversible reduction in LVEF in seven patients. Safety and tolerability similar to placebo.
EXPLORER‐MRI	Prospective, Phase III, double‐blinded, randomized, clinical trial	EXPLORER‐HCM substudy.	*Mavacamten* 2.5–15 mg daily dosed to achieve LVOTG <30 mmHg and serum concentration of 350–700 ng/mL	35	30 weeks	To examine the effect of Mavacamten on cardiac structure and function.	Reduction in LV mass, LV mass index, intracellular myocardial mass index, LV wall thickness, LA volume index, NT‐proBNP, hs‐cTnI. Change in LV mass index was positively correlated with change in hs‐cTnI.	LVEF reduction with Mavacamten was similar to the EXPLORER‐HCM population.
VALOR‐HCM	Prospective, Phase III, randomized, double‐blind, placebo‐controlled trial	NYHA III–IV (or NYHA II with syncope). LVOTG ≥50 mmHg and met criteria for SRT.	*Mavacamten* 5–15 mg daily, titrated up based on LVOTG and LVEF	112	16 weeks	To determine if Mavacamten benefits those who no longer meet guideline criteria or choose to not undergo SRT for HCM.	Improvement of postexercise LVOTG, NYHA function class, KCCQ score, serum NT‐proBNP, and cardiac troponin I.	Minor reduction of LVEF.
MAVA‐LTE	Prospective, open‐label, dose‐blinded	EXPLORER‐HCM patients.	*Mavacamten* 5 mg daily with dose adjustments at Weeks 4, 8, 12, and 24, up to 15 mg daily, based on TTE (if LVOTG ≥30 mmHg)	231	62 weeks	To evaluate long‐term safety, efficacy, and clinically guided dosing.	Improvement in resting and Valsalva LVOTG, NT‐proBNP, NYHA classification.	Minor reduction of LVEF.
REDWOOD‐HCM: Cohort 1	Prospective, Phase II, double‐blinded, randomized, clinical trial	NYHA II or III. Resting LVOTG ≥50 mmHg or resting LVOTG ≥30 mmHg and Valsalva LVOT‐G ≥50 mmHg, LVEF ≥60%.	*Aficamten* 5–15 mg daily	41	10 weeks	To study the safety and tolerability of Aficamten and its association with resting and Valsalva LVOTG, LVEF, NYHA class, and NT‐proBNP.	Statistically significant improvement in resting and Valsalva LVOTG, and NT‐proBNP. Improvement in NYHA classification/symptoms.	Stress cardiomyopathy, back pain. No patients with LVEF <50%.
REDWOOD‐HCM: Cohort 2	Prospective, Phase II, double‐blinded, randomized, clinical trial	NYHA II or III. Resting LVOTG ≥50 mmHg or resting LVOTG ≥30 mmHg and Valsalva LVOTG ≥50 mmHg, LVEF ≥60%.	*Aficamten* 10–30 mg daily	41	10 weeks	To study the safety and tolerability of Aficamten and its association with resting and Valsalva LVOTG, LVEF, NYHA class, and NT‐proBNP.	Statistically significant improvement in resting and Valsalva LVOTG, and NT‐proBNP. Improvement in NYHA classification/symptoms.	Stress cardiomyopathy, back pain. 2 patients with LVEF <50%.
REDWOOD‐HCM: Cohort 3	Prospective, Phase II, open‐label, clinical trial	NYHA II or III. Resting LVOTG ≥30 mmHg and provoked LVOTG ≥50 mmHg.	*Aficamten* 5–15 mg daily in addition to disopyramide (dosing TBD)	13	10 weeks	To assess the safety and efficacy of a cardiac myosin inhibitor in combination with disopyramide.	TBD	TBD
FOREST‐HCM (formerly REDWOOD‐OLE)	Ongoing, prospective, Phase II, open‐label, clinical trial	Patients from parent Aficamten trials.	*Aficamten* (dosing TBD)	54	5 years	To assess changes in cardiac function and morphology.	TBD	TBD
SEQUIOA‐HCM	Prospective, Phase III, double‐blinded, randomized, clinical trial	NYHA II or III. Resting LVOTG ≥30 mmHg and post‐Valsalva LVOT G ≥50 mmHg.	*Aficamten* 5–20 mg daily	270	24 weeks	To primarily assess changes in pVO_2_ while secondary outcomes will assess changes in LVOTG, NYHA functional class, KCCQ and total exercise capacity (CPET).	TBD	TBD
TEMPO	Prospective, Phase III, double‐blinded, randomized, cross‐over clinical trial	NYHA ≥II. Peak LVOTG ≥30 mmHg at rest and/or ≥50 mmHg with provocation.	*Metoprolol* 50, 100, or 150 mg daily	29	Two consecutive 2‐week periods	To investigate the effects of metoprolol on LVOTG, symptoms, and exercise capacity.	Metoprolol reduced LVOT obstruction at rest, peak exercise, postexercise, and during Valsalva. NYHA class and KCCQ‐CSS also improved.	No SAEs.
Subtype: Non‐obstructive hypertrophic cardiomyopathy								
MAVERICK‐HCM	Prospective, Phase II, double‐blinded, randomized, clinical trial	NYHA II or III. LVOTG <30 mmHg.	*Mavacamten* Group 1 target plasma concentration: 200 ng/mL. Group 2: 500 ng/mL	59	16 weeks	To assess the efficacy and safety of Mavacamten in symptomatic nHCM.	Reduction in NT‐proBNP and cTnI.	5 patients experienced LVEF ≤45%.
REDWOOD‐HCM: Cohort 4	Prospective, Phase II, double‐blinded, randomized, clinical trial	NYHA II or III. LVOTG <30 mmHg.	*Aficamten* 5–15 mg daily	30–40	10 weeks	Safety and tolerability of Aficamten and its association with resting and Valsalva LVOTG, LVEF, NYHA class, and NT‐proBNP.	Improved symptoms and cardiac biomarkers.	Well tolerated, no discontinuations due to AE.
RESTYLE‐HCM	Prospective, Phase II, double‐blinded, randomized, clinical trial	NYHA II or III. LVOTG <30 mmHg.	*Ranolazine* 1000 mg BID	80	5 months	Efficacy of Ranolazine in improving the exercise capacity, diastolic function, and symptomatic status.	No differences noted in primary outcomes. Reduced 24‐h PVC burden by ≥50%.	Constipation, vomiting, dizziness, and insomnia.
ODYSSEY‐HCM	Prospective, Phase III, double‐blinded,randomized, placebo‐controlled clinical trial	NYHA Class II or III. LVOTG <30 mmHg at rest and <50 mmHg with provocation.	*Mavacamten*	420	52 weeks	Change from baseline in KCCQ‐23‐CSS and pVO_2_ after 52 weeks.	TBD	TBD
Miscellaneous studies								
VANISH	Prospective, Phase II, double‐blinded, randomized, clinical trial	Patients with pathogenic or likely pathogenic HCM sarcomere mutation based on genetic testing, split into two groups.	*Valsartan* adults: 320 mg daily; children: 80–160 mg daily	178	2 years	Safety and efficacy of the angiotensin II receptor blocker Valsartan in attenuating disease evolution in early HCM.	Valsartan improved a composite score integrating measures of cardiac structure/function and remodeling, cardiac dimensions, LV diastolic velocity via Doppler, and NT‐proBNP levels.	The safety and tolerability of valsartan were similar to placebo.
SESAME	Translational research	5 naive pigs and 5 pigs with percutaneous aortic banding‐induced LV hypertrophy.	*Septal scoring along the midline endocardium using electrosurgery*	10	30 days	To develop a novel transcatheter procedure, mimicking surgical myotomy.	SESAME myotomy along the intended trajectory was achieved in all animals. LVOT area increased, septal thickness decreased along with a reduced distance between the anterior mitral leaflet to septum distance.	Two naive pigs developed ventricular septal defects due to excessively deep lacerations in thin baseline septa. One mitral chord rupture and another developed left axis deviation on ECG.
PIMSRA	Prospective, single‐arm, open‐label, cohort study	NYHA II or higher. Drug‐refractory HCM LVOTG >50 mmHg.	*Septal RF ablation*	200	19 months	To describe the safety and outcomes of PIMSRA in drug‐refractory HCM.	Improvement of NYHA class, 6‐min walking distance, maximum septal thickness, LVOT diameter, mitral valve systolic anterior motion grade, resting and provoked LVOTG, LAVi, and exercise time.	Pericardial effusion requiring intervention (9.5%), permanent RBBB (3.5%), septal branch aneurysm (1.0%), resuscitated ventricular fibrillation (1.0%), 30‐day death (1.0%). Mild worsening of LVEF that was statistically but not clinically significant.

Abbreviations: AE, adverse event; BID, twice daily; CI, confidence interval; CMR, cardiac magnetic resonance imaging; CPET, cardiopulmonary exercise test; cTnI, cardiac troponin I; *E*/*e*′, index that reflects left ventricular filling pressure; ECG, electrocardiogram; HCM, hypertrophic cardiomyopathy; HCMSQ‐SoB, Hypertrophic Cardiomyopathy Questionnaire—Shortness of Breath; hs‐cTnI, high‐sensitivity cTnI; IVS, interventricular septum; KCCQ, Kansas Cardiomyopathy Questionnaire; KCCQ‐CSS, Clinical Summary Score of the Kansas Cardiomyopathy Questionnaire; LA, left atrium; LAVi, left atrial volume index; LV, left ventricle; LVEF, left ventricular ejection fraction; LVOT, left ventricular outflow tract; LVOTG, left ventricular outflow tract gradient; mg, milligrams; nHCM, non‐obstructive hypertrophic cardiomyopathy; NT‐proBNP, N‐terminal pro‐hormone brain natriuretic peptide; NYHA, New York Heart Association; oHCM, obstructive hypertrophic cardiomyopathy; PVC, premature ventricular contraction; pVO_2_, mixed venous oxygen pressure; RBBB, right bundle branch block; RF, radiofrequency; SAE, serious adverse event; SRT, septal reduction therapy; TBD, to be determined; TTE, transthoracic echocardiography.

*MAVACAMTEN; a first‐in‐class, allosteric, reversible inhibitor of B‐cardiac myosin is currently FDA‐approved for treatment of symptomatic oHCM*: Mavacamten inhibits cardiac‐specific myosin ATPase, which decreases myosin‐actin cross bridging as this is an energy‐dependent process.^56^ ATPase inhibition has a positive dose‐dependent relationship and, most importantly, is reversible. Its half‐life is 10 days, administered once daily, and has biliary clearance.^56^ In a murine model harboring heterozygous pathogenic mutations in the cardiac myosin heavy chain, chronic administration of mavacamten suppressed the development of ventricular hypertrophy, cardiomyocyte disarray, and myocardial fibrosis, and attenuated hypertrophic and profibrotic gene expression.^57^ These potent and protean effects support a disease‐modifying potential for CMI. The safety and efficacy of mavacamten have been studied extensively over the last few years (Table 1).

*PIONEER‐HCM* ^58^ was a Phase II, unblinded clinical trial to evaluate the pharmacokinetics, safety, and efficacy of mavacamten. Twenty‐one subjects with NHYA Class II and III symptoms were included in the trial. All participants met the criteria for oHCM (LV wall thickness ≥15 or ≥13 mm with a family history of HCM) with a resting LVOT gradient of ≥30 mmHg or a postexercise gradient ≥50 mmHg. Participants were split into two cohorts and received therapy for 12 weeks. Cohort A (*n* = 11) was given a higher starting dose of mavacamten to demonstrate the dose–response relationship of decrease in myocardial contractility and left ventricular ejection fraction (LVEF). Cohort B (*n* = 10) was started on a lower dose of mavacamten with dose uptitration based on reduction in LVOT gradient to demonstrate the dose–response relationship on gradient improvement. Both cohorts had a reduction in postexercise LVOT gradients, but Cohort A (median dose 15 mg/day) had a greater reduction than Cohort B (5 mg/day); −89.5 mmHg (95% confidence interval [CI], −138.3 to −40.7, *p* = .008) versus −25.0 mmHg (95% CI, −47.1 to −3.0, *p* = .020). Eight participants in Cohort A had a postexercise gradient <30 mmHg, while no participants in Cohort B achieved this. Participants in Cohort A had a greater improvement in pVO_2_ compared to Cohort B (+3.5 mL/kg/min [95% CI, 1.2–5.9] vs. +1.7 mL/kg/min [95% CI, 0.33–3.3]). Both cohorts also had a reduction in serum NT‐proBNP (−425 pg/dL [interquartile range, −748 to −68] in Cohort A and −81 pg/dL [interquartile range, −637 to −16] in Cohort B). The main adverse effect was a drop in resting LVEF that also followed a dose–response relationship (−14.6% [95% CI, −23.1% to −6.2%] in Cohort A and −5.5% [95% CI, −9.8% to −1.2%] in Cohort B). All changes were reversible and returned to baseline 4 weeks after mavacamten was stopped. The authors concluded that mavacamten demonstrated a dose–response relationship for the improvement in LVOT gradients, exercise capacity, wall stress, and a reduction in systolic function. Based on the pharmacodynamics observed in this trial, the authors demonstrated a therapeutic range that maximizes the positive impact of the drug while minimizing the drop in LVEF.

Twelve patients who completed PIONEER‐HCM trials also enrolled in PIONEER‐OLE: a 3‐year open‐label extension study to assess long‐term safety. mavacamten was well tolerated for up to 55 weeks. At 48 weeks, the resting LVOT gradient for all patients was below 50 mmHg. LV EF remained normal for all patients. Reductions in cardiac biomarkers, septal wall thickness, and left atrial size were seen. Of 12 patients, 9 achieved NYHA Class I functional status.^59^

*EXPLORER‐HCM* ^60^ was a Phase III multicenter, randomized, double‐blinded, placebo‐controlled trial that included 251 participants treated with mavacamten (*n* = 123) or placebo (*n* = 128). All participants had oHCM and were classified as NYHA II or III functional class. The total study duration was 30 weeks, and participants had clinical evaluations every 2–4 weeks to evaluate for improvements in LVOT gradients and drop in LVEF; mavacamten doses were increased if there was a suboptimal impact on the former with minimal effect on the latter. The primary endpoint was a composite of ≥1.5 mL/kg per min increase in pVO_2_ and ≥1 NYHA class improvement OR ≥3.0 mL/kg/min increase in pVO_2_ without worsening of NYHA functional status. Secondary endpoints were changes in postexercise LVOTG, pVO_2_, NYHA class, Kansas City Cardiomyopathy Questionnaire (KCCQ) Clinical Summary Score, and Hypertrophic Cardiomyopathy Symptom Shortness of Breath score. Most patients were also being treated with BB or CCB. Disopyramide was prohibited for safety reasons. Among the 123 participants in the mavacamten group, 45 (37%) met either component of the primary endpoint compared to 22 (17%) in the placebo group, a difference of 19.4% (95% CI, 8.7%–30.1%; *p* = .0005). Twenty percent of the mavacamten group met both primary endpoint criteria, compared to only 8% in the placebo group. In addition, the mavacamten group had a greater decrease in postexercise LVOT gradient (−35.6 mmHg [95% CI, −43.2 to 28.1; *p* < .0001]) and an increase in pVO_2_ (1.4 mL/kg/min [95% CI, 0.6–2.1; *p* = .0006]). Twice as many participants in the mavacamten group had ≥1 NYHA class improvement compared to the placebo group (65% vs. 31%, *p* < .0001). One‐third of patients on mavacamten achieved NYHA Class I symptom status and LVOTG <30 mmHg. These benefits were seen at the beginning of the first scheduled follow‐up visit, which occurred in Week 4. Nine participants in the mavacamten group, seven during the study period, and two at the study conclusion had an LVEF that dropped below 50% compared to two in the placebo group. All but one participant in the mavacamten group recovered their LVEF after an 8‐week drug washout period; one participant had a complication related to an AF ablation and had partial recovery of LVEF after a washout period. Cardiac magnetic resonance in substudy analysis (EXPLORER‐MRI) revealed a change in LV mass index of −17.4 g/m^2^ in the mavacamten group versus −1.6 g/m^2^ in the placebo group (*p* < .0001).^61^ In addition, there was a change in LVEF of −6.6% in the mavacamten group versus −0.3% in the placebo group (*p* = .0025). There were 231 of 244 patients who completed treatment in the Phase 3 EXPLORER‐HCM trial that were enrolled in an ongoing, dose‐blinded 5‐year extension study (MAVA‐LTE). Mavacamten was well tolerated, with sustained reductions in LVOT gradients and NT‐proBNP at 84 weeks.^62^

*VALOR‐HCM* ^63^: The promising results of the EXPLORER‐HCM led to the development of the VALOR‐HCM trial, which evaluated the impact of mavacamten on patients who met the criteria for SRT according to the ACC/AHA/European Society of Cardiology guidelines.^64^ This double‐blind, placebo‐controlled, multicenter trial enrolled 112 participants who were followed for 16 weeks. All patients had NYHA III–IV (or NYHA II with syncope) symptoms despite maximally tolerated medical therapy. Participants were randomized to receive either mavacamten or a placebo and had clinical follow‐ups every 4 weeks. The primary endpoint was a composite of a number of participants who remained guideline eligible for SRT or elected for SRT at 16 weeks. After 16 weeks, 18% of participants in the mavacamten group versus 77% of participants in the placebo group (*p* < .001) met the primary endpoint. Two (3.6%) participants in both groups decided to proceed with SRT. For those who did not elect for SRT, 14% in the mavacamten group versus 70% in the placebo group still met the guideline criteria for SRT. Secondary endpoints included change in postexercise LVOT gradient, NYHA function class, score on the KCCQ, serum NT‐proBNP, and cardiac troponin I, all favored the mavacamten group with significant improvements compared to placebo. Two participants in the mavacamten group (3.6%) experienced a drop in LVEF to <50%, necessitating temporary cessation of mavacamten. However, mavacamten was eventually restarted with the restoration of systolic function in these participants without any other adverse events.

In summary, the PIONEER‐HCM, EXPLORER‐HCM, and VALOR‐HCM demonstrated significant molecular and clinical improvements in participants with oHCM and progressive disease. These clinical trials ultimately led to mavacamten becoming the first FDA‐approved medication for oHCM in the Spring of 2022. However, there remains a gap in treating patients with nHCM, which prompted the MAVERICK‐HCM trial.

*MAVERICK‐HCM* ^65^ was a dose‐ranging Phase II, randomized, placebo‐controlled trial that enrolled 59 participants with symptomatic non‐obstructive HCM (NYHA II or III). Forty‐six participants were randomized to three groups: one that received mavacamten at a dose to achieve a target plasma concentration of 200 ng/mL, one at a target concentration of 500 ng/mL, and a third that received a placebo. All patients were on concurrent therapy with BB and CCB. The study's primary analysis was to assess the safety and tolerability of mavacamten. Exploratory analyses evaluated the improvements in cardiac biomarkers, echocardiographic changes, and a composite functional endpoint. NT‐proBNP was lower in both mavacamten groups, with a 47% reduction in the lower plasma concentration group, a 53% reduction in the higher plasma concentration group, and minimal change in the placebo group (*p* = .0005). There was a pooled analysis of both mavacamten groups compared to placebo for cardiac troponin I. There was a 34% reduction in the pooled mavacamten group compared to a slight increase in the placebo group (*p* = .009). Diastolic function was evaluated, observing changes to *E*/*e*′ and *e*′ velocity. There were no echocardiographic changes between the mavacamten groups and the placebo. In addition, a composite functional endpoint mirrored the EXPLORER‐HCM trial; a composite of ≥1.5 mL/kg/min increase in pVO_2_ and improvement in NYHA functional class OR ≥3.0 mL/kg/min or greater increase in pVO_2_ without worsening of NYHA functional status. Initially, there were no differences found among all of the groups. However, when analyzing a subgroup of participants with a baseline cardiac troponin I level >99th percentile and *E*/*e*′ average >14, 33% of participants in the mavacamten group met the primary endpoint, while none of the patients in the placebo group did (*p* = .03). Five participants treated with mavacamten, two in the lower dose concentration and three in the higher cohort, were found to have LVEF <45%. Subgroup analyses from studies that fail to reach significance in the primary endpoint can only be considered hypothesis‐generating. Therefore, a Phase III, double‐blind randomized trial (ODYSSEY_HCM) has now started enrolling symptomatic patients with nHCM.

*Mavacamten in clinical practice*: Mavacamten (Camzyos®) was FDA‐approved in April 2022 to treat adults with symptomatic NYHA Class II–III oHCM to improve functional capacity and symptoms. Mirroring EXPLORER‐HCM data, it is advised to use baseline medical therapy with BB and CCB, and to use mavacamten as a next‐tier agent. Its use requires certification for the REMS program (Risk Evaluation and Mitigation Strategy) to detect HF due to systolic dysfunction with periodic echocardiograms and allow for screening of drug interactions before each dispense. Although several practices have quickly adopted this program, having a trained support staff is key to meeting the program's logistical demands.

*AFICAMTEN*, like mavacamten, is a next‐generation CMI intended to reduce cardiac contractility but has a shorter half‐life reaching a steady state within 2 weeks and a narrower concentration–response profile. It does not show substantial CYP induction and demonstrates a wide therapeutic window, unlike mavacamten.^66^

*REDWOOD‐HCM* ^67^: This multicenter, randomized, double‐blind, placebo‐controlled Phase II clinical trial assessed the safety, pharmacokinetics, and tolerability of aficamten in 95 participants. The initial trial was over 10 weeks, with an extension study that will extend to 5 years (REDWOOD‐HCM OLE). All participants with HCM had NYHA II or III functional class and LVOT obstruction. The primary outcome was to evaluate for adverse effects, while secondary outcomes included changes in LVOT gradients, NYHA class, and KCCQ scores. In the aficamten group, it was noted that resting and Valsalva LVOT gradients showed improvement starting at 2 weeks. At weeks 12 and 24, there were reductions in resting gradient compared to baseline (mean change (SD) = −32.6 mmHg (28), *p* < .0001 at 12 weeks, −32.8 mmHg (32.3), *p* = .0003 at 24 weeks), and Valsalva gradient (−42.7 mmHg (38.7), *p* < .0001 at 12 weeks, −51.1 mmHg (35.3), *p* < .0001 at 24 weeks). Compared to the baseline, 61% of patients improved by one NYHA class, and 17% improved by two classes (*p* < .0001). At 24 weeks, the aficamten group had improvements in all domains of the KCCQ. There was only a small decrease in LVEF; (−3.2% (4.2), *p* = .0038 at 24 weeks). No patients had LVEF <50%. Of note, the first two cohorts of patients were concurrently treated with BB and CCB, and the third cohort was concurrently treated with Disopyramide and BB. There were substantial reductions in LVOTG and biomarkers along with improvement in symptoms were seen in all groups. After the promising results of the initial REDWOOD cohorts in oHCM, patients with nHCM in REDWOOD Cohort 4 showed that aficamten improved symptoms and cardiac biomarkers in 41 patients.^68^

*FOREST‐HCM*, is an ongoing open‐label extension study for eligible patients with oHCM and nHCM who completed a parent study of aficamten, enrolled 45 patients to date. Aficamten was well tolerated and sustained its effects for up to 48 weeks. Of 19 patients meeting standard criteria for SRT at baseline, none met those criteria at 48 weeks.^69^

*SEQUOIA‐HCM* is a Phase III multicenter, randomized, double‐blind, placebo‐controlled trial of aficamten that is currently enrolling participants to become the largest HCM trial to date. The primary outcome will assess changes in pVO_2_, while secondary outcomes will assess changes in LVOT gradients, NYHA functional class, KCCQ, and total exercise capacity. There is much anticipation for the results of the SEQUOIA trial with the goal of another FDA‐approved medication for oHCM. Further analysis may also assess its use in patients with nHCM.

### 4.5 SESAME: Novel interventional therapy to treat LVOTO

In 2022, Lederman et al. published the results of a preclinical study demonstrating the feasibility of a novel transcatheter myotomy procedure called Septal Scoring Along the Midline Endocardium (SESAME)^70^ (Table 1). In this study, the septal myocardium was lacerated using transcatheter electrosurgery. The procedure was successfully performed on 10 pigs under fluoroscopic and intracardiac echocardiographic guidance. The results demonstrated reduced septal thickness, increased LVOT area, and anterior mitral leaflet to septum distance. During follow‐up, the SESAME laceration did not appear to propagate, cardiac function did not deteriorate, and all animals maintained normal coronary perfusion after the study. Moreover, none of the animals developed heart block. Complications included VSD in two animals and mitral chord rupture in one animal. Subsequently, a first‐in‐human case report showed a successful SESAME procedure.^71^ Currently, SESAME is for compassionate use only and is yet to be FDA‐approved.

## 5 CONCLUSIONS AND FUTURE DIRECTIONS

HCM is a long‐recognized disorder that can result in severe symptoms and life‐altering complications such as HF and SCD. The advent of CMIs as targeted therapy for treating LVOTO in oHCM represents an exciting step forward. Prospective, randomized trials have shown mavacamten to be effective and safe in relieving symptoms, and they can potentially defer SRT in a subset of oHCM patients, leading to FDA approval. Phase 3 trials on aficamten have completed enrollment; promising data may lead to the approval of a second CMI in oHCM patients. Several long‐term clinical trials are underway and are expected to shed further light on the effect of CMIs in nHCM. Furthermore, HCM is a heterogenous disease in many ways, from its diverse disease‐causing mutations, clinical presentation, outcomes, and management. Therefore, one can foresee the importance of a multidisciplinary HCM team approach that brings different specialties together to provide patients with the best evidence‐based practices and tailored individual treatment plans.

## CONFLICT OF INTEREST STATEMENT

The authors declare no conflict of interest.

## Data Availability

Data sharing is not applicable to this article as no new data were created or analyzed in this study.
